# Novel peritoneal catheters with silver particles lower rates of peritonitis in patients with cirrhosis and refractory ascites

**DOI:** 10.1371/journal.pone.0359408

**Published:** 2026-10-06

**Authors:** Sarah Lisa Schütte, Benjamin Heidrich, Heiner Wedemeyer, Benjamin Maasoumy, Tammo Lambert Tergast

**Affiliations:** 1 Hannover Medical School, Department of Gastroenterology, Hepatology, Infectious Diseases and Endocrinology, Hannover, Germany; 2 German Center for Infection Research (DZIF), Hannover-Braunschweig, Germany; Cleveland Clinic, UNITED STATES OF AMERICA

## Abstract

**Background:**

Treatment options for refractory ascites (RA) in cirrhosis are scarce. When TIPS insertion is contraindicated, tunneled peritoneal catheters (cPeCa) offer an alternative treatment. However, cPeCa are associated with high incidences of infection-related complications. Novel PeCa embedded with silver particles (sPeCa) have been introduced to address this issue.

**Methods:**

We evaluated all patients with cirrhosis and RA treated with PeCa or ascites pump at Hannover Medical School from 2012–2023. Analysis was conducted utilizing a multivariable Competing-Risk-Analysis and afterwards a 3:1-Propensity-Score-Matching (PSM) to adjust for potential differences. Last, sPeCa-patients were compared to those with ascites pump. Study endpoints were mortality, peritonitis, device explantation and rehospitalization.

**Results:**

150 patients with cPeCa were compared to 27 sPeCa-patients. Treatment with sPeCa was associated with significantly reduced incidences of peritonitis (HR 0.28, p = 0.002), device explantation (HR 0.37, p = 0.03) and rehospitalization (HR 0.48, p = 0.02). After PSM, 81 cPeCa-patients were compared to 27 sPeCa-patients. Mortality was comparable (HR 1.66, p = 0.29). Patients with sPeCa experienced less peritonitis (HR 0.34, p = 0.008), explantation (HR 0.39, p = 0.04) and rehospitalization (HR 0.52, p = 0.046). When compared to ascites-pump, sPeCa-patients demonstrated similar rates of mortality (HR 0.95, p = 0.94), peritonitis (HR 0.97, p = 0.96) and explantation (HR 0.89, p = 0.88), while rehospitalization rate was significantly higher in ascites pump (HR 2.83, p = 0.02).

**Conclusion:**

Treatment with sPeCa is associated with less infection-associated complications and could therefore be a superior therapeutic approach compared to cPeCa.

## Introduction

Onset of refractory ascites (RA) is the hallmark of end stage decompensated advanced chronic liver disease (dACLD). As mortality rates increase, there is a concomitant decrease in quality of life, compounded by limited treatment options. Liver transplantation (LTx) remains inaccessible to a most patients due to organ shortage. Transjugular intrahepatic portosystemic shunt (TIPS) placement has emerged as a highly efficacious therapeutic approach for RA. Nonetheless, certain patients are not eligible for TIPS insertion due to contraindications like heart failure, pulmonary hypertension or recurrent hepatic encephalopathy without identifiable triggers [1]. Currently, guidelines recommend repeated large volume paracenteses (LVP) with albumin infusion as the standard of care (SOC) in these patients. However, treatment with tunneled peritoneal catheters (PeCa) offers an alternative therapeutic approach. These catheters enable home-based therapy, aiming to reduce the frequency of hospital admissions and alleviating the financial burden of repeated LVP on the healthcare system. Some previous studies have investigated device associated complications and reasons for device explantations [2]. Here, peritonitis was a major complication with reported incidences of up to 58% and the most frequent reason for device explantation [3]. Likewise, one study indicated a higher risk of peritonitis compared to patients with cirrhosis that received ascites pump [4]. Moreover, infections are a leading cause for mortality in individuals with dACLD. Furthermore, these patients are highly susceptible for infections. Hence, minimizing the risk for infections is essential in the clinical management of this vulnerable collective. In this context, silver particles have previously been associated with antimicrobial features [5,6]. The addition of silver particles to the silicone of the PeCa could therefore lead to decreased bacterial colonialization of the device and subsequently to a lower incidence of infections. PeCa with the addition of silver particles (sPeCa) are commercially available for recent years. However, there are no studies available that compare the incidence of infections or the need for device explantations in patients with sPeCa to those receiving treatment with other low-flow devices, such as ascites pumps or conventional PeCa (cPeCa).

Hence, this study compared the incidence of important clinical endpoints in patients with RA ineligible for TIPS which either received cPeCa, sPeCa or ascites pump.

## Methods

### Study cohort

Data of this retrospective cohort study was collected between 2012 and 2023 at Hannover Medical School, Germany. All consecutive patients treated for RA who underwent either cPeCa, sPeCa or ascites pump implantation were evaluated for this study. In total, 469 patients were considered. Exclusion criteria consisted of missing ACLD or RA, underlying malignant diseases, prior organ transplantation, age below 18 years, short life expectancy, ongoing fulminant complications or missing data on implantation (S1 Fig). Patients not matching those criteria were included in this study.

5 patients have been treated with TIPS prior to implantation. Since RA persisted, PeCa implantation was conducted afterwards. The 18 µm sized silver particles of the sPeCa are embedded into the silicon polymers. After all procedures, the particles are firmly anchored into the material.

The catheters were placed in the Endoscopy unit of Hannover Medical School. Prior to implantation, patients received a single-shot antibiotic infusion (Ampicillin/Sulbactam). After locating a suitable puncture site using ultrasound guidance, the catheter was inserted and tunneled up to 10 cm through subcutaneous tissue from where the tip was placed in the peritoneal cavity. The procedure was performed unter local anesthesia or sedation, based on the patients’ preference. The daily drainage was performed by the patients themselves, their relatives or dedicated health care services. Patients and relatives are educated on proper hygienic practice before management of the catheter [3].

Prior to implantation, all patients are evaluated in a multidisciplinary conference to allow for individualized treatment strategies. If patients fulfil contraindications against TIPS insertion, they are offered the implantation of a low-flow device or, at their request, repeated large volume paracenteses with albumin infusions.

### Data assessment and study endpoints

Data regarding study patients and laboratory values were gathered automatically by the Clinical Information System and subsequently validated manually. Information on the patients’ clinical course was acquired from their medical records between 01.08.2023 and 31.07.2024. Patients were followed for one year after device implantation. Presence of RA was defined according to current guidelines [1]. In this study, endpoints included Liver transplant (LTx)-free survival and incidences of peritonitis, device explantation and rehospitalization. Additionally, secondary endpoints included bacteremia, septicemia, urinary tract infections (UTI) and acute-on-chronic liver failure (ACLF). Peritonitis was defined as the presence of ≥250 polymorphnuclear cells in the ascites fluid or culture-positive ascitic fluid with a cellcount <250 polymorphnuclear cells. Peritonitis within the first seven days post-implantation was considered procedure-related and excluded from analysis. ACLF was defined according to the CLIF-C-OF criteria [1].

### Study design

Three analyses were conducted:

First, all patients that received cPeCa were compared to those receiving sPeCa. Multivariable competing risk analyze models included six variables. Presence of diabetes mellitus type 2 and history of spontaneous bacterial peritonitis (SBP) were included, as they are risk factors for development of SBP [7,8]. Norfloxacin intake was included to adjust for preventive medication. Additionally, proton pump inhibitor (PPI) intake has previously been associated with higher risk of SBP and therefore also included [9]. Moreover, model for endstage liver disease (MELD) score was included in our model to adjust for the severity of the liver disease.

Afterwards, a 3:1 Propensity Score Matching (PSM) was conducted to adjust for potential differences in baseline characteristics. Matching factors included MELD and history of SBP. After PSM, 108 patients were included in the further analysis, of which 27 received sPeCa and the remaining 81 patients were treated with a cPeCa.

Last, univariable and multivariable competing risk analyses comparing 27 patients with sPeCa to 38 patients with an ascites pump were performed. Due to smaller sample sizes in the ascites pump cohort and the sPeCa group and therefore less events of interest, the multivariable model was reduced to MELD score, age and history of SBP [8].

### Statistics

The data underwent analysis utilizing SPSS Version 28 (IBM) and R Version 4.3.2 (R Project for Statistical Computing, packages MatchIt, Rcmdr, Rcmdr.Plugin EZR, CompRisk) [10,11]. Nearest neighbor matching with a caliper set at 0.25 was used to perform PSM. Matching factors included MELD, to adjust for the severity of the liver disease and history of SBP. To evaluate whether PSM was successful, standardized mean differences (SMD) were utilized. SMD below 0.1 were considered to indicate successful PSM (S1 Table). All categorical variables are presented in total numbers and proportions. In the overall unmatched cohorts Chi-square test and Fisher’s exact test were applied to calculate group differences, while Cochran-Mantel-Haenszel-Test was applied in the matched cohort. Continuous variables are presented as mean with standard deviation (SD). Analysis of continuous variables was conducted utilizing unpaired Student’s t-test in the unmatched cohorts and ANOVA in the matched cohort. Longitudinal values were analyzed utilizing paired t-test and were presented as mean with SD. Survival incidences were illustrated with Kaplan-Meier curves in the analyses. Univariable Fine and Gray’s Competing Risk Analysis were conducted in all cohorts, while the unmatched cohorts were additionally analyzed in multivariable competing risk analyses. Death and LTx were considered as competing events. Regarding mortality, LTx was the only competitor while death was the event of interest.

### Ethics

This study followed the Declaration of Helsinki. It was approved by the local ethics committee (Ethics approval number Nr. 9428_BO_K_2020) [12]. All patients declared their written informed consent for participation in this study and analysis of the data.

## Results

### Analysis I

Of all 177 included patients, 150 (85%) received cPeCa, whilst 27 (15%) patients were treated with sPeCa (S1 Fig). The majority of patients were male (cPeCa: n = 105 (70%), sPeCa: n = 20 (74%), p = 0.67) and those in the sPeCa group were numerically older (cPeCa: 59 [11] years, sPeCa: 64 [13] years, p = 0.07). Patients with cPeCa had significantly higher bilirubin (cPeCa: 3.35 [4.96] mg/dl, sPeCa: 1.67 [1.83] mg/dl, p = 0.002). Still, MELD score did not differ between the cohorts (cPeCa: 18 [6] points, sPeCa: 16 [5] points, p = 0.15). The daily drained amount of ascites was higher in the cPeCa group (cPeCa: 1.9 [0.89] l, sPeCa: 1.2 [0.5] l, p < 0.001). The predominant contraindication for TIPS insertion was risk for hepatic encephalopathy (HE) in the cPeCa cohort, while only n = 8 (30%) of sPeCa patients could not undergo TIPS insertion due to risk of HE. In this group, most patients were contraindicated for TIPS insertion because of impaired liver function and therefore had a high risk of hepatic decompensation after TIPS insertion (sPeCa: n = 9 (33%), cPeCa: n = 1 (1%), p < 0.001) (Table 1). Norfloxacin intake was comparable in both cohorts (sPeCa: n = 19 (70%), cPeCa: n = 91 (63%), p = 0.45; Table 1).

**Table 1:** Baseline characteristics prior to PSM.

Parameter	PeCa (n = 150)	sPeCa (n=27)	p-value
Male/Female, No. (%)	105 (70)/ 45 (30)	20 (74)/ 7 (26)	0.67
Underlying disease			
ALD, No. (%)	54 (36)	9 (33)	0.79
Met-ALD, No. (%)	16 (11)	6 (22)	0.09
MALSD, No. (%)	17 (11)	2 (7)	0.74
Viral, No. (%)	20 (13)	1 (4)	0.21
Cryptogenic, No. (%)	14 (9)	4 (15)	0.49
Other, No. (%)	30 (20)	5 (19)	0.86
Age, mean (SD), y	59 (11)	64 (13)	0.07
MELD, mean (SD)	18 (6)	16 (5)	0.15
Creatinine, mean (SD), mg/dL	1.69 (0.84)	1.76 (0.76)	0.68
Bilirubin, mean (SD), mg/dL	3.35 (4,96)	1.67 (1,83)	0.002
INR, mean (SD)	1.45 (0.30)	1.37 (0.30)	0.24
Leukocyte count, mean (SD), × 10^3^/μL	7.02 (4.95)	6.57 (4.23)	0.66
Sodium, mean (SD), mmol/L	134 (7)	132 (6)	0.24
ALT, mean (SD), IU/mL	36 (47)	28 (25)	0.39
Albumin, mean (SD), g/L	30 (7)	30 (8)	0.69
Platelet count, mean (SD), × 10^3^/μL	111 (89)	111 (68)	0.99
Daily drained amount of ascites, mean (SD), L	1.9 (0.89)	1.2(0.5)	<0.001
History of SBP, No. (%)	91 (61)	9 (33)	0.008
Medication			
Norfloxacin, No. (%)	91 (63)	19 (70)	0.45
Diabetes mellitus Type 2, No. (%)	39 (26)	6 (22)	0.68
PPI, No. (%)	127 (85)	18 (69)	0.05
NSBB, No. (%)	47 (31)	9 (33)	0.84
Diuretics, No. (%)	88 (60)	17 (63)	0.79
Contraindications against TIPS			
Risk for HE, No. (%)	89 (59)	8 (30)	0.004
Heart failure, No. (%)	27 (18)	6 (22)	0.60
Technically impossible, No. (%)	12 (8)	2 (7)	1.00
Impaired liver function, No. (%)	2 (1)	9 (33)	<0.001
Other, No. (%)	20 (13)	3 (11)	1.00

Abbreviations: ALD, alcohol-associated liver disease; ALT, alanine amino transferase; HE, hepatic encephalopathy; INR, international normalized ratio; MASLD, metabolic dysfunction-associated steatotic liver disease; MELD, Model of endstage liver disease; MetALD, metabolic and alcohol-associated liver disease; PeCa, tunneled peritoneal catheter; NSBB, non-selective beta blockers; PPI, proton pump inhibitors; PSM, propensity score matching; SBP, spontaneous bacterial peritonitis; sPeCa, tunneled peritoneal catheter with silver particles; TIPS, transjugular intrahepatic portosystemic shunt.

Univariable competing risk analysis indicated a similar risk of mortality between patients with cPeCa and sPeCa (HR 1.20, 95% CI 0.53–2.70, p = 0.67). The risk for device explantation and rehospitalization within the first year after device implantation were numerically decreased in those with sPeca (HR 0.43, 95% CI 0.18–0.99, p = 0.05; HR 0.58, 95% CI 0.32–1.05, p = 0.07; Fig 1A, Fig 1C-D). Patients who received sPeCa treatment were at a lower risk of peritonitis (HR 0.27, 95% CI 0.13–0.58, p < 0.001; Fig 1B). No differences were found in the risks of bacteremia, septicemia or UTI (HR 0.61, 95% CI 0.14–2.61, p = 0.05; HR 0.80, 95% CI 0.10–6.63, p = 0.84; HR 0.16, 95% CI 0.02–1.12, p = 0.08; respectively). The risk of progression of ACLF did not differ between patients treated with cPeCa and those treated with sPeCa (HR 1.19, 95% CI 0.73–1.94, p = 0.48). The number of events can be found in S1A Table. Multivariable competing risk analysis indicated a significantly lower incidence of rehospitalization, device explantation and peritonitis (subdistributed HR 0.48, 95% CI 0.25–0.91, p = 0.02; subdistributed HR 0.37, 95% CI 0.15–0.90, p = 0.03; subdistributed HR 0.28, 95% CI 0.13–0.62, p = 0.002, respectively, Table 2), while there was no association between sPeCa implantation and mortality (subdistributed HR 1.25, 95% CI 0.50–3.12, p = 0.64; Table 2).

**Table 2:** Multivariable competing risk analysis models of PeCa compared to sPeCa.

Parameter	Hazard ratio	Lower 95% CI	Upper 95% CI	p-value
Mortality				
Diabetes mellitus type 2	1.54	0.75	3.16	0.24
MELD	1.11	1.04	1.171	<0.001
Norfloxacin intake	1.07	0.52	2.22	0.85
PPI intake	0.85	0.34	2.11	0.73
Treatment with sPeCa	1.25	0.50	3.12	0.64
History of SBP	0.71	0.34	1.48	0.36
Peritonitis				
Diabetes mellitus type 2	0.94	0.57	1.55	0.81
MELD	1.01	0.97	1.05	0.76
Norfloxacin intake	0.83	0.52	1.32	0.44
PPI intake	0.94	0.51	1.72	0.83
Treatment with sPeCa	0.28	0.13	0.62	0.002
History of SBP	1.34	0.84	2.13	0.22
Device explantation				
Diabetes mellitus type 2	0.78	0.41	1.48	0.45
MELD	0.97	0.93	1.01	0.13
Norfloxacin intake	1.02	0.59	1.76	0.95
PPI intake	0.96	0.44	2.07	0.92
Treatment with sPeCa	0.37	0.15	0.90	0.03
History of SBP	0.90	0.55	1.48	0.68
Rehospitalization				
Diabetes mellitus type 2	0.85	0.54	1.35	0.50
MELD	0.97	0.94	1.01	0.10
Norfloxacin intake	0.76	0.51	1.13	0.18
PPI intake	1.04	0.62	1.74	0.88
Treatment with sPeCa	0.48	0.25	0.91	0.02
History of SBP	1.00	0.69	1.46	0.99

Abbreviations: CI, confidence interval; MELD, Model of endstage liver disease; PPI, proton pump inhibitors; SBP, spontaneous bacterial peritonitis; sPeCa, tunneled peritoneal catheter with silver particles

**Fig 1:**
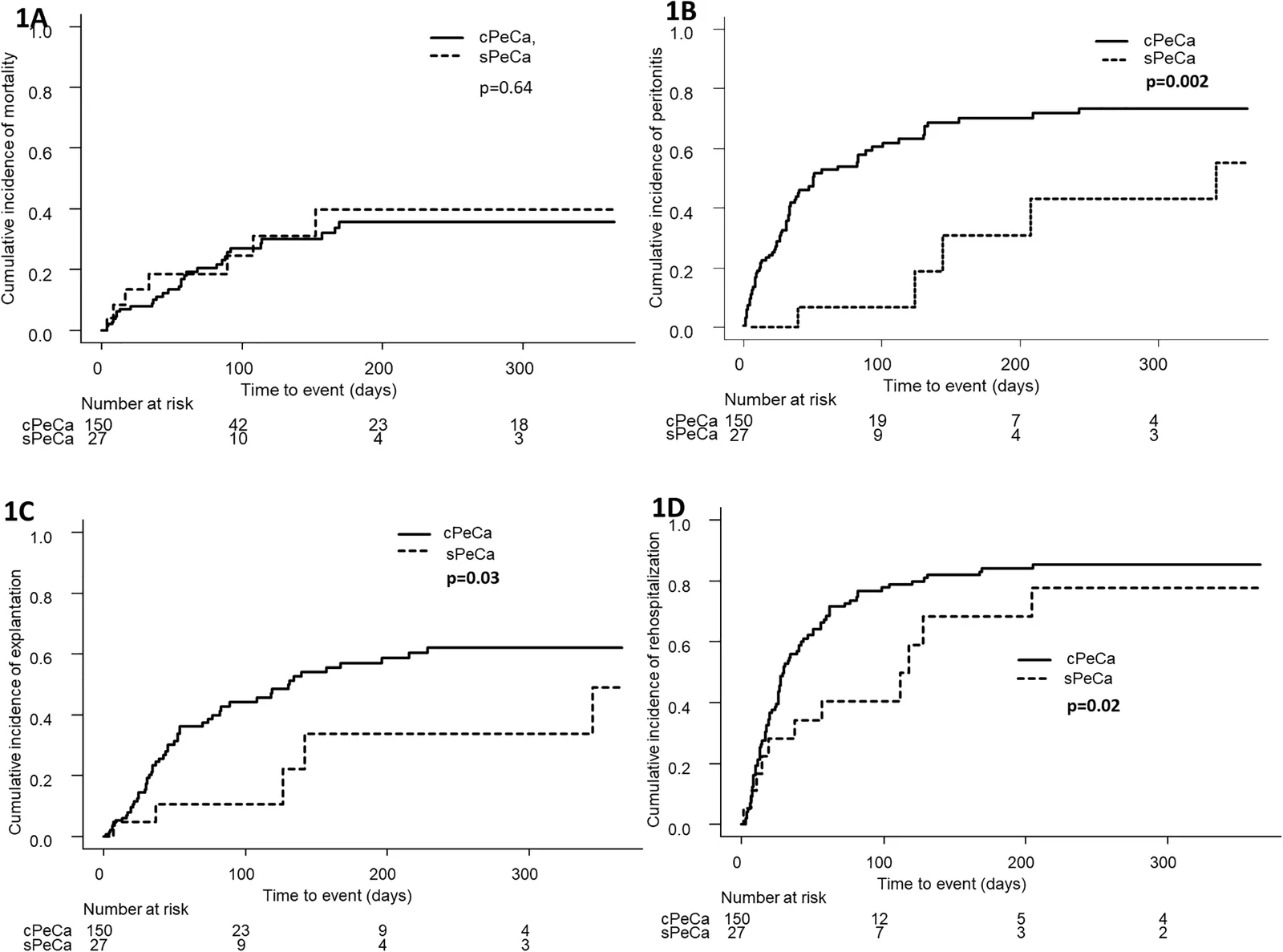
Kaplan-Meier-curves illustrating the cumulative incidences of mortality (A), peritonitis (B), device explantation (C) and rehospitalization (D) in patients with cPeCa compared to those with sPeCa in one year. Death and LTx were considered as competing events. Attached p-values are the result of multivariable competing risk analysis. Abbreviations: cPeCa, conventional tunneled peritoneal catheter; sPeCa, tunneled peritoneal catheter with silver particles.

One month after implantation, MELD score decreased in the overall cohort from 17.6 points (SD 6 points) to 18.4 points (SD 5.8 points) (p = 0.03). Albumin levels decreased from 29.9 g/l (SD 7.47 g/l) to 26.29 g/l (SD 5.83 g/l) (p < 0.001). Creatinine values increased from 1.70 mg/dl (SD 0.82 mg/dl) prior to implantation to 2.21 mg/dl (SD 1.24 mg/dl) (p < 0.001) after one month.

### Analysis II

Afterwards, PSM was conducted. SMDs were below 0.1 after PSM and therefore matching was considered successful (MELD: 0.02, History of SBP: 0.00; S2 Table, S2 Fig). A total of n = 108 patients remained for further analysis. Most patients were male (cPeCa: n = 59 (73%), sPeCa: n = 20 (74%), p = 0.90) and the MELD scores in both cohorts did not differ (cPeCa: 16 [5] points, sPeCa: 16 [5] points, p = 0.91). The number of patients with a history of SBP was comparable across both cohorts (cPeCa: n = 27 (33%), sPeCa: n = 9 (33%), p = 1.00) (Table 3). Again, Norfloxacin intake was similar in both cohorts (sPeCa: n = 19 (70%), cPeCa: n = 47 (59%), p = 0.40; Table 3). Mortality did not differ in the first year after implantation (HR 1.66, 95% CI 0.65–4.22, p = 0.29; Fig 2A). However, patients with sPeCa had a significantly lower risk of peritonitis, device explantation and rehospitalization (HR 0.34, 95% CI 0.16–0.76, p = 0.008; HR 0.39, 95% CI 0.16–0.95, p = 0.04 and HR 0.52, 95% CI 0.27–0.99, p = 0.046; respectively; Fig 2B-2D) within one year. The number of events can be found in S1B Table. A positive cultural germ detection was successful in 24 out of 30 documented cases of peritonitis in cPeCa (with 43 identified pathogens) and in 3 out of 5 cases of peritonitis in sPeCa (with 6 identified pathogens) in patients with sPeCa (S3 Table). Staphylococcus spp. emerged as the most prevalent genus in both groups (PeCa: n = 20 (47%); sPeCa: n = 5 (83%)), with S. epidermidis being the predominant species (PeCa: n = 11 (27%); sPeCa: n = 3 (50%)) (S3 Table).

**Table 3:** Baseline characteristics after PSM.

Parameter	PeCa (n = 81)	sPeCa (n=27)	p-value
Male/Female, No. (%)	59 (73)/ 22 (27)	20 (74)/ 7 (26)	0.90
Underlying disease			
ALD, No. (%)	29 (36)	9 (33)	1.00
Met-ALD, No. (%)	9 (11)	6 (22)	0.26
MASLD, No. (%)	10 (12)	2 (7)	0.73
Viral, No. (%)	11 (14)	1 (4)	0.29
Cryptogenic, No. (%)	7 (9)	4 (15)	0.58
Other, No. (%)	16 (20)	5 (19)	0.89
Age, mean (SD), y	61 (11)	64 (13)	0.29
MELD, mean (SD)	16 (5)	16 (5)	0.91
Creatinine, mean (SD), mg/dL	1.57 (0.62)	1.76 (0.76)	0.19
Bilirubin, mean (SD), mg/dL	2.28 (2.16)	1.67 (1.83)	0.19
INR, mean (SD)	1.38 (0.28)	1.37 (0.30)	0.85
Leukocyte count, mean (SD), × 10^3^/μL	6.76 (4.26)	6.57 (4.23)	0.85
Sodium, mean (SD), mmol/L	134 (7)	132 (6)	0.24
ALT, mean (SD), IU/mL	35 (41)	28 (25)	0.40
Albumin, mean (SD), g/L	30 (7)	30 (8)	0.92
Platelet count, mean (SD), × 10^3^/μL	109 (83)	112 (68)	0.91
Daily drained amount of ascites, mean (SD), L	1.8 (0.8)	1.2 (0.5)	0.002
History of SBP, No. (%)	27 (33)	9 (33)	1.00
Medication			
Norfloxacin, No. (%)	47 (59)	19 (70)	0.40
Diabetes mellitus Type 2, No. (%)	23 (28)	6 (22)	0.71
PPI, No. (%)	68 (84)	18 (69)	0.18
NSBB, No. (%)	25 (31)	9 (33)	1.00
Diuretics, No. (%)	54 (68)	17 (63)	0.85
Contraindications against TIPS			
Risk for HE, No. (%)	48 (59)	8 (30)	0.015
Heart failure, No. (%)	19 (24)	5 (19)	0.79
Technically impossible, No. (%)	2 (3)	2 (7)	0.56
Impaired liver function, No. (%)	1 (1)	9 (33)	<0.001
Other, No. (%)	11 (14)	3 (11)	1.00

Abbreviations: ALD, alcohol-associated liver disease; ALT, alanine amino transferase; HE, hepatic encephalopathy; INR, international normalized ratio; MASLD, metabolic dysfunction-associated steatotic liver disease; MELD, Model of endstage liver disease; MetALD, metabolic and alcohol-associated liver disease; NSBB, non-selective beta blockers; PeCa, tunneled peritoneal catheter; PPI, proton pump inhibitors; PSM, propensity score matching; SBP, spontaneous bacterial peritonitis; sPeCa, tunneled peritoneal catheter with silver particles; TIPS, transjugular intrahepatic portosystemic shunt

**Fig 2:**
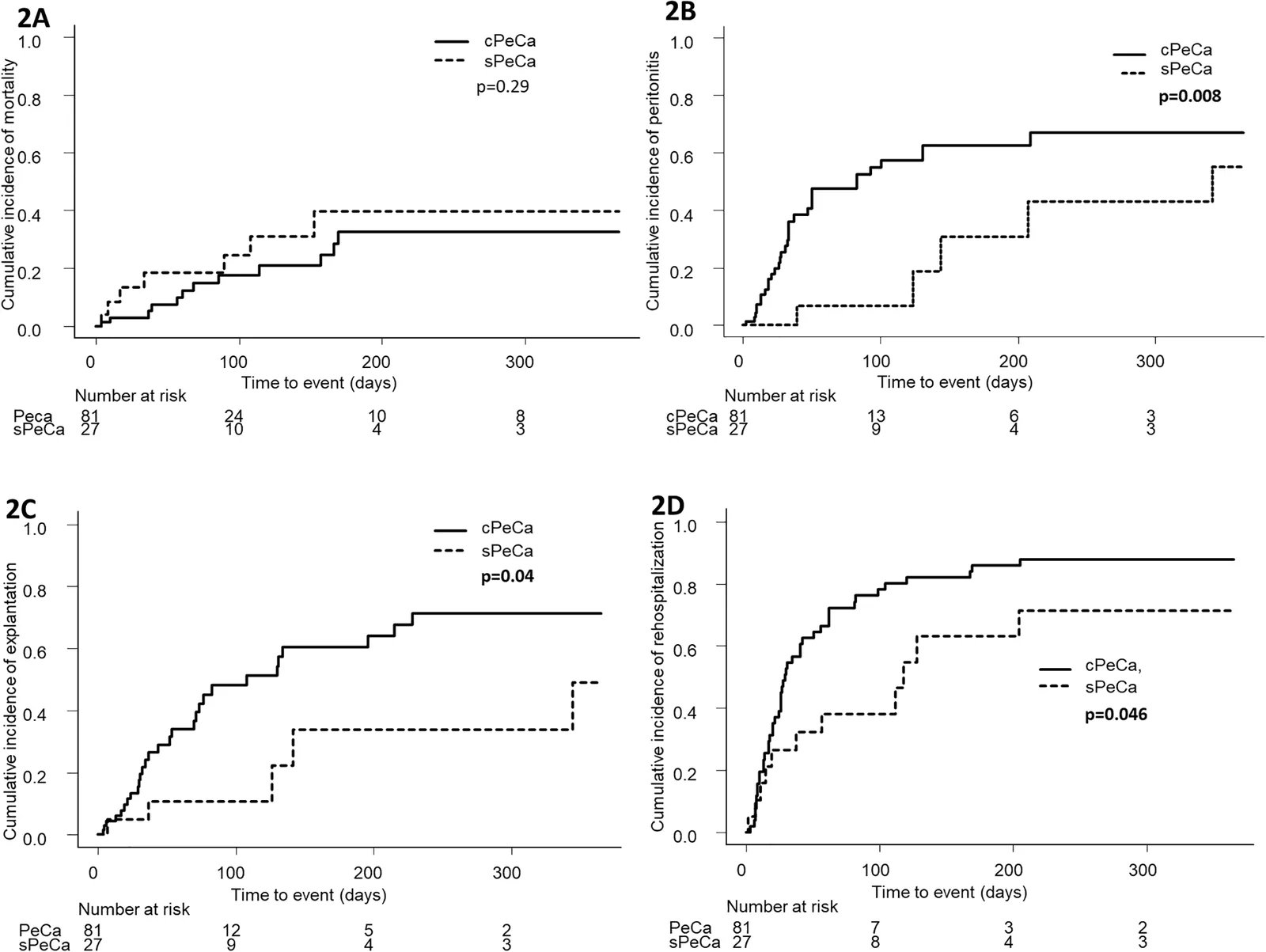
Kaplan-Meier-curves illustrating the cumulative incidences of mortality (A), peritonitis (B), device explantation (C) and rehospitalization (D) in patients with cPeCa compared to those with sPeCa in one year after PSM. Death and LTx were considered as competing events. Attached p-values are the result of univariable competing risk analysis. Abbreviations: cPeCa, conventional tunneled peritoneal catheter; PSM, propensity score matching; sPeCa, tunneled peritoneal catheter with silver particles.

Patients treated with cPeCa demonstrated numerically higher explantation rates compared to those receiving sPeCa (cPeCa: n = 33 (41%), sPeCa: n = 7 (26%)). The majority of cPeCa were explanted due to infections of the catheter, followed by transplantation and dysfunction of the catheter (n = 19 (58%), n = 4 (12%) and n = 3 (9%), respectively; S4 Table). In the sPeCa cohort, infection remained the primary cause for explantation, followed by transplantation and dysfunction of the device (n = 3 (43%), n = 2 (29%) and n = 1 (14%), respectively; S4 Table). Patients receiving sPeCa therapy demonstrated numerically reduced rates of rehospitalization compared to their counterparts treated with cPeCa (cPeCa: n = 45 (55%), sPeCa: n = 12 (45%); S5 Table). While peritonitis accounted for the majority of rehospitalizations in patients treated with cPeCa (cPeCa: n = 12 (15%), sPeca: n = 3 (11%)), kidney impairment was the predominant reason for readmission in sPeCa-treated patients (cPeCa: n = 9 (11%), sPeCa: n = 5 (19%) (S5 Table). Notably, n = 3 (4%) patients with cPeCa were rehospitalized due to peritonitis and HE and n = 1 patient was readmitted due to peritonitis and kidney impairment, while no patient with sPeCa was hospitalized because of peritonitis and an associated complication (S5 Table).

### Analysis III

Additionally, we compared 27 patients with sPeCa to 38 patients with ascites pump. The majority of patients were male (sPeCa: n = 20 (74%), ascites pump: n = 26 (68%), p = 0.62). Patients with sPeCa were older (sPeCa: 64 [13] years, ascites pump 56 [12] years, p = 0.01) and had higher MELD score (sPeCa: 16 [5] points, ascites pump: 13 [4], p = 0.008) (S6 Table). No difference regarding administration of Norfloxacin has been observed (sPeCa: n = 19 (70%), ascites pump: n = 28 (74%), p = 0.77; S6 Table). In univariable analysis, no differences regarding mortality (HR 0.51, 95% CI 0.19–1.36, p = 0.18), peritonitis (HR 0.83, 95%CI 0.30–2.32, p = 0.72), explantation (HR 0.62, 95% CI 0.21–1.79, p = 0.37) and rehospitalization (HR 1.78, 95% CI 0.91–3.48, p = 0.09) were observed between both cohorts (Fig 3). However, after adjustment for MELD, age and history of SBP, patients with ascites pump had a significantly higher risk of rehospitalization compared to those with sPeCa (subdistributed HR 2.83, 95% CI 1.20–6.68, p = 0.02; Table 4, Fig 3). Still, neither mortality nor peritonitis nor explantation rates differed between both cohorts (subdistributed HR 0.95, 95% CI 0.27–3.35, p = 0.94; subdistributed HR 0.97, 95% CI 0.26–3.53, p = 0.96; subdistributed HR 0.89, 95% CI 0.19–24.16, p = 0.88; respectively, Table 4, Fig 3). The number of events is given in S1C Table.

**Table 4:** Multivariable competing risk analysis models of sPeCa compared to ascites pump.

Parameter	Hazard ratio	Lower 95% CI	Upper 95% CI	p-value
Mortality				
Age	1.05	0.99	1.11	0.06
MELD	1.17	1.00	1.37	0.05
Treatment with sPeCa	0.95	0.27	3.35	0.94
History of SBP	0.23	0.04	1.40	0.11
Peritonitis				
Age	1.01	0.97	1.06	0.65
MELD	1.06	0.94	1.19	0.37
Treatment with sPeCa	0.97	0.26	3.53	0.96
History of SBP	0.95	0.31	2.93	0.92
Device explantation				
Age	1.05	0.99	1.10	0.07
MELD	1.04	0.90	1.20	0.57
Treatment with sPeCa	0.89	0.19	4.16	0.88
History of SBP	0.83	0.24	2.86	0.77
Rehospitalization				
Age	1.02	0.99	1.04	0.27
MELD	1.14	1.04	1.25	0.007
Treatment with sPeCa	2.83	1.20	6.68	0.02
History of SBP	0.73	0.36	1.48	0.38

Abbreviations: CI, confidence interval; MELD, Model of endstage liver disease; SBP, spontaneous bacterial peritonitis; sPeCa, tunneled peritoneal catheter with silver particles

**Fig 3:**
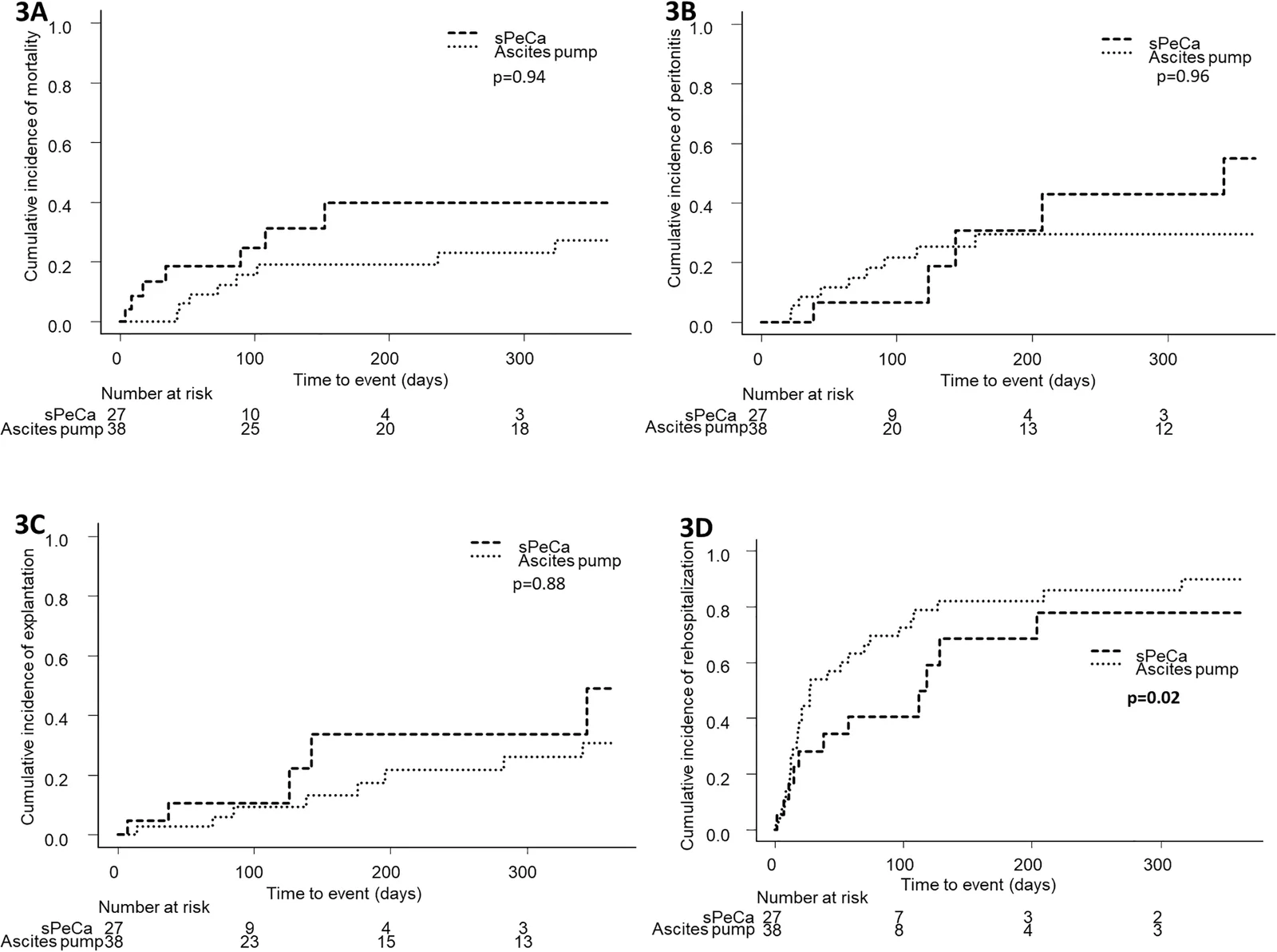
Kaplan-Meier-curves illustrating the cumulative incidences of mortality (A), peritonitis (B), device explantation (C) and rehospitalization (D) in patients with cPeCa compared to those with sPeCa in one year. Death and LTx were considered as competing events. Attached p-values are the result of multivariable competing risk analysis. Abbreviations: cPeCa, conventional tunneled peritoneal catheter; sPeCa, tunneled peritoneal catheter with silver particles.

## Discussion

The development of RA is a hallmark in the natural course of ACLD. While treatment of RA is challenging, TIPS insertion has emerged to achieve effective symptom control for many patients. However, few patients are not eligible for this procedure due to contraindications or do not experience resolution of ascites despite TIPS insertion [1]. While treatment with PeCa aims to enable home-based ascites management in these vulnerable patients, it has been linked to high incidences of peritonitis and infection-related complications leading to frequent rehospitalizations [4]. To address this risk, PeCa with embedded silver particles have been introduced. This is the first study that compared these novel sPeCa to cPeCa and ascites pump. Treatment with sPeCa was linked to significantly lower incidences of peritonitis, device explantation or readmissions.

The formation of biofilms on catheters is a significant factor contributing to their colonization, particularly when the devices are not utilized with the utmost care and attention. By embedding silver particles into the silicone polymer, bacterial growth can be significantly reduced [13]. Different modes of action have been described how silver molecules exert antimicrobial effects such as binding to functional groups of bacterial enzymes, inhibiting mitosis or release of potassium from the cytoplasm of bacteria [14]. While no studies have previously investigated the efficacy of silver particles in the setting of tunneled ascites catheters, numerous studies examined the effect of silver-coating on a range of other devices [15]. Kollef et al. observed significantly lower incidences of ventilation-associated pneumonia in patients treated with silver-coated endotracheal tubes compared to uncoated tubes [16]. Furthermore, they also observed a prolonged time to development of ventilation-associated pneumonia. Other studies associated silver-coating of joint prosthesis with lower rates of prosthetic joint infections [17]. These findings are well in line with our study since we also observed lower rates of device infection and infection related complications.

Besides palliative care in patients with progressed dACLD, PeCa are also used as a bridge-to-transplant concept in selected patients suffering from RA and awaiting LTx. In both scenarios, the idea behind PeCa insertion is to achieve a high quality of life and to avoid frequent admissions due to the need for LVP. Some prior studies compared patients undergoing PeCa placement with SOC, i.e., repeated LVP with albumin infusion. Interestingly, mortality and peritonitis rates were comparable [3,18]. Patients with dACLD are highly susceptible for bacterial infections due a compromised immune function, well known as cirrhosis-associated immune dysfunction [19]. Still, rehospitalization rates were high in patients with PeCa and were frequently associated with infections due to treatment with the device. Thus, the idea of lower rehospitalizations by device implantation was not demonstrated in a real-life cohort [3]. Meanwhile, sPeCa treatment was associated with a lower incidence of rehospitalization compared to that with cPeCa. Therefore, one could hypothesize that treatment with sPeCa may be associated with lower rates of peritonitis compared to patients treated with SOC. However, these effects need to be investigated in future prospective studies. Both devices are available for the same price, at least in Germany. Hence, based on our data sPeCa should be implanted instead of cPeCa, if both options are available.

Overall, mortality rates did not show significant differences between patients treated with cPeCa and those with sPeCa, despite their lower incidence of peritonitis. Mortality in the setting of dACLD and RA is high with around 50% and infections are a key driver of disease progression and mortality [20,21]. Hence, one could expect a lower mortality in those with sPeCa. Peritonitis in patients with PeCa can be accompanied by purulent ascites in their daily paracentesis or redness of the puncture site. Hence, patients could have been alerted early and presented therefore quite early for medical advice. Furthermore, detection of the infection is possible fast since ascites can be drawn from the device immediately without any time loss [22]. These two factors contribute to an early diagnosis and lead to an early start of antibiotic treatment. This could be one reason for similar mortality rates in this study. Furthermore, it is currently unknown, if the established cut-off of 250PMN/µl which is used for diagnosis of SBP is appropriate to diagnose and treat device-associated infections. Presence of the device could also induce sterile inflammation. Hence, sensitivity and specificity for the diagnosis of peritonitis could be impaired. Therefore, life-threatening peritonitis could have been anticipated and treated while, in fact, sterile inflammation was present. This would explain why we did not observe a higher mortality, since the analysis might have compared patients without actual infection but a sterile inflammation. Still, the amount of inflammation seems to be lower in those with sPeCa as PMN counts were not as frequently increased compared to the cPeCa, which is indicated by the lower peritonitis rates in this study.

Another option to treat RA in TIPS ineligible patients is the implantation of an ascites pump, which was also associated with a lower peritonitis incidence compared to PeCa in another study from our center. However, this device needs additional surgery under general anesthesia and drainage volume can only be adjusted via experienced physicians in specialized centers. Moreover, explantation also requires surgery under general anesthesia. Both PeCa and sPeCa can be implanted with only local anesthesia and explantation can be done in almost every hospital using only local disinfection and a scalpel, which is favorable in patients with end stage liver disease, since general anesthesia comes at a significant risk of further decompensation and ACLF [23]. Last, ascites pumps are currently not available in Europe. In our analysis, we did not observe differences in the incidences of peritonitis or device explantation. However, patients undergoing treatment with an ascites pump experienced significantly higher readmission rates. Given that one primary goal of using devices in RA is to reduce frequent hospitalizations, treatment with sPeCa may offer an alternative to ascites pump therapy.

This study faces important limitations. One of the main limitations are small samples sizes, especially in the sPeCa cohort. Despite best efforts to ensure highest data quality, adverse events or complications could potentially have gone undetected due to the retrospective study design. A higher number of patients with cPeCa had a history of SBP and are conclusively at higher risk to develop another episode. Hence, to adjust for this risk factor, we it into the multivariable model and into the matching. However, it still could potentially influence the incidences of peritonitis. The sPeCa have been introduced relatively recently to the market while in past years only cPeCa oder ascites pumps have been available. Therefore, non-contemporaneous control bias may underlie, since we are comparing cohorts from different years. Of note, standard protocols on the handling of the catheters have not changed and remained same through all years and for different catheters.

In conclusion, this study implicates that addition of silver molecules to cPeCa is associated with a lower risk of device infection, peritonitis and rehospitalization in patients with dACLD and RA. Nonetheless, prospective studies are needed to confirm these findings and to investigate further endpoints such as quality of life or cost efficacy.

### Lay summary

1. Summarize the established knowledge on this subject
  - Treatment with tunneled peritoneal catheters in patients not eligible for placement of a transjugular intrahepatic portosystemic shunt is associated with high risk of infections.
  - Novel tunneled peritoneal catheters embedded with silver particles have been introduced to address this infection risk.
2. What are the significant and/or new findings of this study?
  - Treatment with tunneled peritoneal catheters embedded with silver particles is associated with significantly lower incidences of infection and infection-related complications compared to conventional tunneled peritoneal catheters and ascites pump.
  - Tunneled peritoneal catheters embedded with silver particals could potentially offer a superior therapeutic approach compared to conventional catheters or ascites pump.

## Supporting information

S1 Table (A-C). Number of events regarding the respective study endpoints in Analysis I-III. Numbers are given in total and proportion. Abbrevations: cPeCa, conventional tunneled peritoneal catheter; sPeCa, tunneled peritoneal catheter with silver particles.
(DOCX)

S2 Table Standardized mean differences before and after PSM. Abbreviations: MELD, model of endstage liver disease; PSM, propensity score matching; SBP, spontaneous bacterial peritonitis; Std., standardized.
(DOCX)

S3 Table Pathogens detected in ascitic fluid during peritonitis within one year after device implantationin analysis II. Abbreviations: PeCa, tunneled peritoneal catheter; sPeCa, tunneled peritoneal catheter with silver particles; PSM, propensity score matching.
(DOCX)

S4 Table Reasons for explantation within one year after device implantation in analysis II. Abbreviations: PeCa, tunneled peritoneal catheter; sPeCa, tunneled peritoneal catheter with silver particles; PSM, propensity score matching.
(DOCX)

S5 Table Reasons for rehospitalization within one year after device implantation in analysis II. The percentages are presented as a proportion of the total cohort. Abbreviations: PeCa, tunneled peritoneal catheter; sPeCa, tunneled peritoneal catheter with silver particles; HE, hepatic encephalopathy; PSM, propensity score matching.
(DOCX)

S6 Table Baseline characteristics of sPeCa compared to ascites pump. Abbreviations: ALD, alcohol-associated liver disease; ALT, alanine amino transferase; HE, hepatic encephalopathy; INR, international normalized ratio; MASLD, metabolic dysfunction-associated steatotic liver disease; MELD, Model of endstage liver disease; MetALD, metabolic and alcohol-associated liver disease; NSBB, non-selective beta blockers; PPI, proton pump inhibitors; SBP, spontaneous bacterial peritonitis; sPeCa, tunneled peritoneal catheter with silver particles; TIPS, transjugular intrahepatic portosystemic shunt.
(DOCX)

S1 Fig Consort figure and exclusion criteria. This figure provides information on patient selection and exclusion criteria from this study.
(PDF)

S2 Fig Distribution of propensity scores of cPeCa and sPeCa after PSM. Shown is a jitter plot of the distribution of propensity scores prior to and after PSM. Abbreviations: cPeCa, conventional tunneled peritoneal catheter; sPeCa, tunneled peritoneal catheter with silver particles; PSM, propensity score matching.
(PDF)

S1 Dataset Minimal anonymized data set. (XLSX)

## Abbreviations

dACLD: decompensated advanced chronic liver disease
CI: confidence interval
cPeCa: conventional tunneled peritoneal catheters
HE: hepatic encephalopathy
HR: hazard ratio
LTx: liver transplantation
LVP: large-volume paracentesis
MELD: model for endstage liver disease
PeCa: tunneled peritoneal catheter
PMN: polymorph nuclear cells
PPI: proton pump inhibtors
PSM: propensity score matching
RA: refractory ascites
SBP: spontaneous bacterial peritonitis
SD: standard deviation
SMD: standardized mean difference
SOC: standard of care
sPeCa: tunneled peritoneal catheters embedded with silver particles
Spp.: species
TIPS: transjugular intrahepatic portosystemic shunt
