# Supplementary material for: Novel peritoneal catheters with silver particles lower rates of peritonitis in patients with cirrhosis and refractory ascites
Source: PLoS One. 2026 Oct 6;21(10):e0359408. doi: 10.1371/journal.pone.0359408 (PMC13641377; doi:10.1371/journal.pone.0359408)
Supplement: Supplementary file 2 [file pone.0359408.s008.pdf]

Figure S2

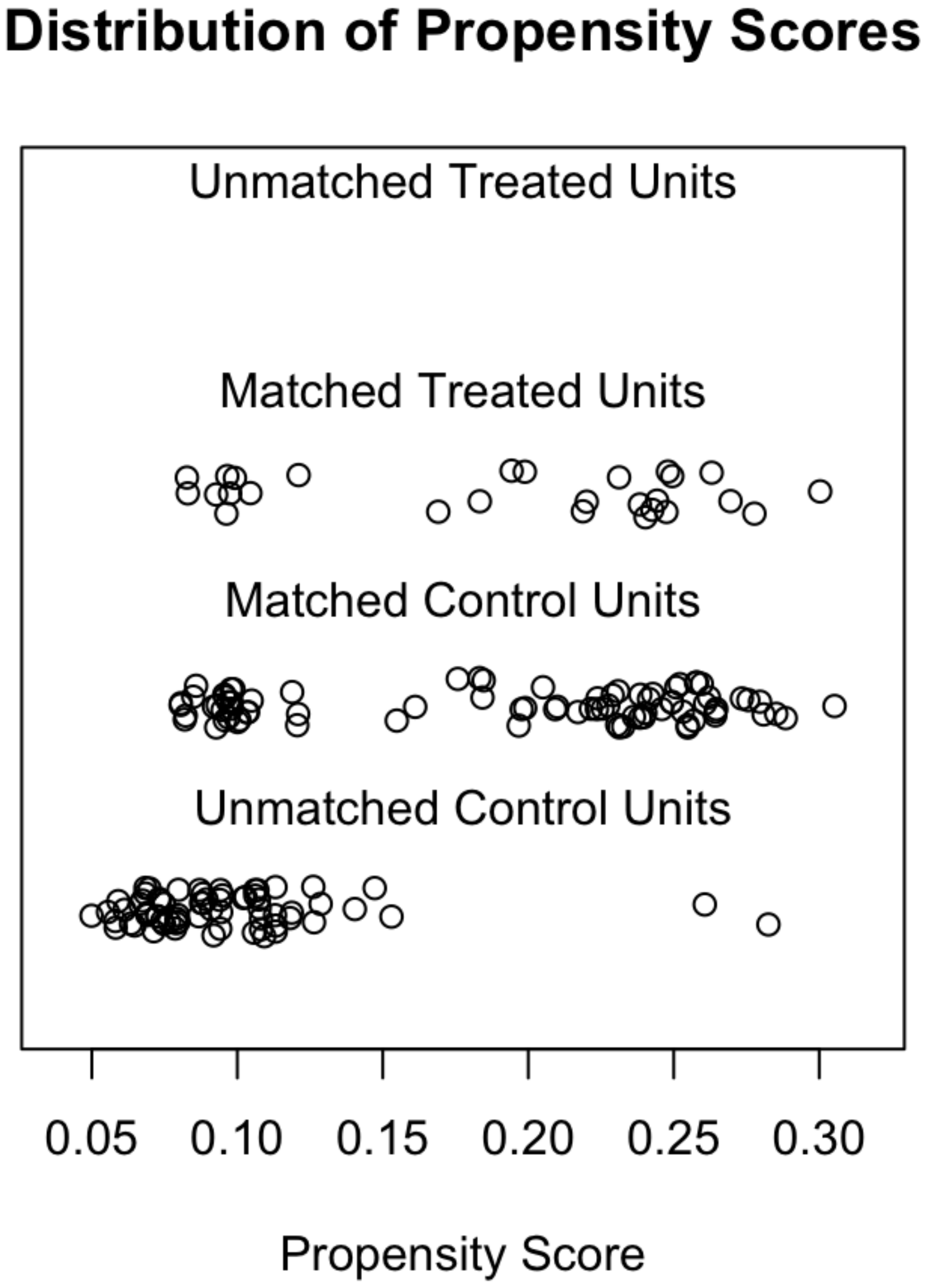

Distribution of propensity scores of cPeCa and sPeCa after PSM
Abbreviations: cPeCa, conventional tunneled peritoneal catheter; sPeCa, tunneled peritoneal catheter with silver particles; PSM, Propensity Score Matching
