# Supplementary material for: Novel peritoneal catheters with silver particles lower rates of peritonitis in patients with cirrhosis and refractory ascites
Source: PLoS One. 2026 Oct 6;21(10):e0359408. doi: 10.1371/journal.pone.0359408 (PMC13641377; doi:10.1371/journal.pone.0359408)
Supplement: Supplementary file 3 [file pone.0359408.s001.docx]

Table S1A:

| Study endpoint | cPeCa (n=150) | sPeCa (n=27) |
| --- | --- | --- |
| Mortality | 31 (21%) | 7 (26%) |
| Peritonitis | 75 (50%) | 5 (19%) |
| Device explantation | 55 (37%) | 5 (19%) |
| Rehospitalization | 83 (55%) | 11 (41%) |
| Liver transplantation | 16 (11%) | 2 (7%) |
| Bacteremia | 20 (13%) | 2 (7%) |
| Septicemia | 8 (5%) | 1 (4%) |
| Urinary tract infection | 34 (23%) | 1 (4%) |
| Acute-on-chronic liver failure | 82 (55%) | 15 (56%) |

Number of events regarding the respective study endpoints in Analysis I. Numbers are given in total and proportion.
Abbrevations: cPeCa, conventional tunneled peritoneal catheter; sPeCa, tunneled peritoneal catheter with silver particles

Table S1B:

| Study endpoint | cPeCa (n=81) | sPeCa (n=27) |
| --- | --- | --- |
| Mortality | 12 (15%) | 7 (26%) |
| Peritonitis | 32 (40%) | 5 (19%) |
| Device explantation | 29 (36%) | 5 (19%) |
| Rehospitalization | 45 (56%) | 11 (41%) |
| Liver transplantation | 9 (11%) | 2 (7%) |

Number of events regarding the respective study endpoints in Analysis II. Numbers are given in total and proportion.
Abbrevations: cPeCa, conventional tunneled peritoneal catheter; sPeCa, tunneled peritoneal catheter with silver particles

Table S1C:

| Study endpoint | sPeCa (n=27) | Ascites pump (n=38) |
| --- | --- | --- |
| Mortality | 7 (26%) | 8 (21%) |
| Peritonitis | 5 (19%) | 9 (24%) |
| Device explantation | 5 (19%) | 8 (21%) |
| Rehospitalization | 11 (41%) | 29 (76%) |

Number of events regarding the respective study endpoints in Analysis III. Numbers are given in total and proportion.
Abbrevations: sPeCa, tunneled peritoneal catheter with silver particles
