# Supplementary material for: Novel peritoneal catheters with silver particles lower rates of peritonitis in patients with cirrhosis and refractory ascites
Source: PLoS One. 2026 Oct 6;21(10):e0359408. doi: 10.1371/journal.pone.0359408 (PMC13641377; doi:10.1371/journal.pone.0359408)
Supplement: Supplementary file 4 [file pone.0359408.s002.docx]

Table S2:

| Parameter | Std. mean difference before PSM | Std. mean difference after PSM |
| --- | --- | --- |
| MELD, per point | -0.32 | 0.02 |
| History of SBP, yes | -0.57 | 0.00 |

Standardized mean differences before and after PSM
Abbreviations: MELD, model of endstage liver disease; PSM, propensity score matching; SBP, spontaneous bacterial peritonitis; Std., standardized
