# Supplementary material for: Novel peritoneal catheters with silver particles lower rates of peritonitis in patients with cirrhosis and refractory ascites
Source: PLoS One. 2026 Oct 6;21(10):e0359408. doi: 10.1371/journal.pone.0359408 (PMC13641377; doi:10.1371/journal.pone.0359408)
Supplement: Supplementary file 5 [file pone.0359408.s003.docx]

Table S3:

| Pathogen | PeCa (n=47) | sPeCa (n=6) |
| --- | --- | --- |
| Staphylococcus spp., n (%) | 20 (43) | 5 (83) |
| Staphylococcus epidermidis, n (%) | 12 (26) | 3 (50) |
| Staphylococcus haemolyticus, n (%) | 6 (13) | 2 (33) |
| Staphylococcus hominis, n (%) | 1 (2) | 0 (0) |
| Staphylococcus aureus, n (%) | 1 (2) | 0 (0) |
| Staphylococcus gordinii, n (%) | 1 (2) | 0 (0) |
| Staphylococcus capitis, n (%) | 1 (2) | 0 (0) |
| Enterococcus spp., n (%) | 12 (26) | 0 (0) |
| Enterococcus faecalis, n (%) | 6 (13) | 0 (0) |
| Enterococcus faecium, n (%) | 3 (6) | 0 (0) |
| Enterococcus avium, n (%) | 2 (4) | 0 (0) |
| Enterococcus gallinarium, n (%) | 1 (2) | 0 (0) |
| Acinetobacter spp., n (%) | 3 (6) | 0 (0) |
| Acinetobacter beijerinckii, n (%) | 1 (2) | 0 (0) |
| Acinetobacter baumanii, n (%) | 1 (2) | 0 (0) |
| Acinetobacter pitii, n (%) | 1 (2) | 0 (0) |
| Acinetobacter calcoaceticus-baumanii complex, n (%) | 1 (2) | 0 (0) |
| Candida spp., n (%) | 1 (2) | 0 (0) |
| Candida albicans, n (%) | 1 (2) | 0 (0) |
| Escherichia spp., n (%) | 2 (4) | 0 (0) |
| Escherichia coli, n (%) | 2 (4) | 0 (0) |
| Streptococcus spp., n (%) | 2 (4) | 0 (0) |
| Streptococcus pneumoniae, n (%) | 1 (2) | 0 (0) |
| Streptococcus dysagalactiae, n (%) | 1 (2) | 0 (0) |
| Corynebacterium spp., n (%) | 0 (0) | 1 (17) |
| Corynebacterium striatum, n (%) | 0 (0) | 1 (17) |
| Enterobacter spp., n (%) | 1 (2) | 0 (0) |
| Enterobacter cloacae, n (%) | 1 (2) | 0 (0) |
| Aerococcus spp., n (%) | 1 (2) | 0 (0) |
| Aeococcus urinae, n (%) | 1 (2) | 0 (0) |
| Pseudomonas spp., n (%) | 1 (2) | 0 (0) |
| Pseudomonas aeruginosa, n (%) | 1 (2) | 0 (0) |
| Klebsiella spp., n (%) | 1 (2) | 0 (0) |
| Klebsiella pneumoniae, n (%) | 1 (2) | 0 (0) |

Pathogens detected in ascetic fluid during peritonitis within one year after device implantation after PSM
Abbreviations: PeCa, tunneled peritoneal catheter; sPeCa, tunneled peritoneal catheter with silver particles; PSM, propensity score matching
