# Supplementary material for: Novel peritoneal catheters with silver particles lower rates of peritonitis in patients with cirrhosis and refractory ascites
Source: PLoS One. 2026 Oct 6;21(10):e0359408. doi: 10.1371/journal.pone.0359408 (PMC13641377; doi:10.1371/journal.pone.0359408)
Supplement: Supplementary file 6 [file pone.0359408.s004.docx]

Table S4:

| Reason for explantation | cPeCa (n=33) | sPeCa (n=7) |
| --- | --- | --- |
| Infection of catheter, n (%) | 19 (58) | 3 (43) |
| Dysfunction of catheter, n (%) | 3 (9) | 1 (14) |
| Transplantation, n (%) | 4 (12) | 2 (29) |
| other, n (%) | 7 (21) | 1 (14) |

Reasons for explantation within one year after device implantation after PSM
Abbreviations: PeCa, tunneled peritoneal catheter; sPeCa, tunneled peritoneal catheter with silver particles; PSM, propensity score matching
