# Supplementary material for: Novel peritoneal catheters with silver particles lower rates of peritonitis in patients with cirrhosis and refractory ascites
Source: PLoS One. 2026 Oct 6;21(10):e0359408. doi: 10.1371/journal.pone.0359408 (PMC13641377; doi:10.1371/journal.pone.0359408)
Supplement: Supplementary file 7 [file pone.0359408.s005.docx]

Table S5:

| Reason for rehospitalization | cPeCa (n=45/81 (55%)) | sPeCa (n=12/27 (45%)) |
| --- | --- | --- |
| Peritonitis, n (%) | 12/81 (15) | 3/27 (11) |
| HE, n (%) | 5/81 (6) | 1/27 (4) |
| Kidney impairment, n (%) | 9/81 (11) | 5/27 (19) |
| HE and kidney impairment, n (%) | 0/81 (0) | 1/27 (4) |
| Peritonitis and HE, n (%) | 3/81 (4) | 0/27 (0) |
| Peritonitis and kidney impairment, n (%) | 1/81 (1) | 0/27 (0) |
| other, n (%) | 15/81 (18) | 2/27 (7) |

Reasons for rehospitalization within one year after device implantation after PSM
The percentages are presented as a proportion of the total cohort.
Abbreviations: PeCa, tunneled peritoneal catheter; sPeCa, tunneled peritoneal catheter with silver particles; HE, hepatic encephalopathy; PSM, propensity score matching
