# Supplementary material for: Novel peritoneal catheters with silver particles lower rates of peritonitis in patients with cirrhosis and refractory ascites
Source: PLoS One. 2026 Oct 6;21(10):e0359408. doi: 10.1371/journal.pone.0359408 (PMC13641377; doi:10.1371/journal.pone.0359408)
Supplement: Supplementary file 8 [file pone.0359408.s006.docx]

Table S6:

| Parameter | sPeCa (n=27) | Ascites pump (n=38) | p-value |
| --- | --- | --- | --- |
| Male/Female, No. (%) | 20 (74) / 7 (26) | 26 (68) / 12 (32) | 0.62 |
| Underlying disease |  | | |
| ALD, No. (%) | 9 (33) | 18 (47) | 0.26 |
| Met-ALD, No. (%) | 6 (22) | 3 (8) | 0.15 |
| MALSD, No. (%) | 2 (7) | 4 (11) | 1.00 |
| Viral, No. (%) | 1 (4) | 6 (16) | 0.22 |
| Cryptogenic, No. (%) | 4 (15) | 0 (0) | 0.03 |
| Other, No. (%) | 5 (19) | 7 (18) | 1.00 |
| Age, mean (SD), y | 64 (13) | 56 (12) | 0.01 |
| MELD, mean (SD) | 16 (5) | 13 (4) | 0.008 |
| Creatinine, mean (SD), mg/dL | 1.76 (0.76) | 1.40 (0.42) | 0.02 |
| Bilirubin, mean (SD), mg/dL | 1.67 (1.83) | 1.49 (1.48) | 0.67 |
| INR, mean (SD) | 1.37 (0.30) | 1.26 (0.17) | 0.10 |
| Leukocyte count, mean (SD), ×10^3^/μL | 6.57 (4.23) | 5.93 (2.38) | 0.44 |
| Sodium, mean (SD), mmol/L | 132 (6) | 135 (6) | 0.15 |
| ALT, mean (SD), IU/mL | 28 (25) | 26 (14) | 0.70 |
| Albumin, mean (SD), g/L | 30 (8) | 28 (6) | 0.15 |
| Platelet count, mean (SD), ×10^3^/μL | 112 (68) | 142 (90) | 0.14 |
| History of SBP, No. (%) | 9 (33) | 10 (26) | 0.54 |
| Medication |  | | |
| Norfloxacin, No. (%) | 19 (70) | 28 (74) | 0.77 |
| Contraindications against TIPS |  | | |
| Risk for HE, No. (%) | 8 (30) | 21 (55) | 0.04 |
| Heart failure, No. (%) | 5 (19) | 3 (8) | 0.20 |
| Technically impossible, No. (%) | 2 (7) | 5 (13) | 0.69 |
| Impaired liver function, No. (%) | 9 (33) | 1 (3) | 0.001 |
| Other, No. (%) | 3 (11) | 7 (18) | 0.50 |
|  |  |  |  |
|  |  |  |  |
|  |  |  |  |

Baseline characteristics of sPeCa compared to ascites pump
Abbreviations: ALD, alcohol-associated liver disease; ALT, alanine amino transferase; HE, hepatic encephalopathy; INR, international normalized ratio; MASLD, metabolic dysfunction-associated steatotic liver disease; MELD, Model of endstage liver disease; MetALD, metabolic and alcohol-associated liver disease; NSBB, non-selective beta blockers; PPI, proton pump inhibitors; SBP, spontaneous bacterial peritonitis; sPeCa, tunneled peritoneal catheter with silver particles; TIPS, transjugular intrahepatic portosystemic shunt
